# Editorial: Resilience of native grass species under climate dynamics: physio-ecological insights

**DOI:** 10.3389/fpls.2026.1973618

**Published:** 2026-09-03

**Authors:** Eduardo Augusto Dias de Oliveira, Weizhou Xu, Bingcheng Xu

**Affiliations:** 1 School of Agriculture, Food and Ecosystem Sciences, Faculty of Science, The University of Melbourne, Parkville, VIC, Australia; 2 College of Advanced Agricultural Sciences, Yulin University, Yulin, China; 3 State Key Laboratory of Soil and Water Conservation and Desertification Control, Northwest A&F University, Yangling, China

**Keywords:** abiotic stress, intraspecific variation, leaf gas films, native grasses, population genomics, provenance, stay-green

## 1 Introduction

Grasslands, which include open grassland, savanna, shrubland, planted pasture and tundra, cover approximately 30 million km^2^ of the terrestrial land surface and support nearly 1 billion livelihoods worldwide (MacDougall et al., 2026). Native grasses carry adaptations to changing environments over tens of millions of years, through the Miocene and subsequent Quaternary cycles (Edwards et al., 2010; Strömberg, 2011). However, grasslands are among the most transformed and least protected terrestrial systems (Bardgett et al., 2021).

This Research Topic sought studies on physiological and ecological strategies for climate stress tolerance, resilience and adaptation; the genetic mechanisms underlying resistance to multiple stresses; and community responses to climate variability, with implications for the conservation and restoration of native grasslands. Four original research articles were accepted, addressing trait-based adaptability along an elevational gradient, chlorophyll catabolism as a regulatory target, a leaf-level response to an extreme hydrological event, and the genetic structure of a wild germplasm resource. All four contributions are set in continental Asian systems, spanning an alpine plateau, a temperate steppe, a subtropical riparian drawdown zone, and an arid dune landscape. Results from these specific sites do not transfer automatically elsewhere, but the questions are universal. These include the importance of matching seed source to site, which is central to restoration worldwide (Broadhurst et al., 2008), the occurrence of leaf gas films across European and Australasian flood gradients (Colmer and Pedersen, 2008; Winkel et al., 2016) and the long-standing use of the stay-green trait in temperate forage-grass breeding (Thomas and Ougham, 2014).

## 2 Retaining function under stress, and for how long

Submergence removes the aerial gas phase on which terrestrial grasses depend. *Cynodon dactylon* leaves were found to be superhydrophobic and retained thin gas films underwater, maintaining higher oxygen partial pressure at the leaf surface for approximately 7 days before the films disappeared Xia et al., within the range reported across species (Winkel et al., 2016). During this period, plants retaining the films kept more stomata open, accumulated more leaf carbohydrates, retained more green leaves, and elongated stems faster. However, three limitations should be noted: first, a surfactant removed the films; second, the water was held near air saturation rather than at the hypoxic levels typical of field floodwater (Bailey-Serres et al., 2012); and finally, the metabolic effects were inferred from metabolite pools rather than measured flux. Together, these results show that a leaf-surface trait can regulate early flood performance.

During darkness, the key trait is the ability to retain chlorophyll and other components of photosynthetic function. The gray-green ecotype of *Leymus chinensis* lost 20% of its chlorophyll over 6 days of darkness compared with 70% in the yellow-green ecotype, and maintained a maximum quantum yield of photosystem II of 0.68, while the yellow-green type fell to 0.23 Naren et al. Rubisco, thylakoid complexes, and crude protein were also preserved in the gray-green ecotype, indicating a functional rather than a cosmetic stay-green phenotype (Thomas and Howarth, 2000). The genes *Non-Yellow Coloring 1 (NYC1)* and *NYC1-like (NOL)*, which are involved in chlorophyll breakdown, showed weaker transcript-level induction in the gray-green ecotype, although the two ecotypes also differed in initial chlorophyll content (5.2 versus 3.6 mg g^-1^ fresh weight). The original *Festuca pratensis* stay-green phenotype came from a breeding population (Thomas and Stoddart, 1975); the presence of a similar phenotype in a wild steppe grass adds to this record. Both studies describe temporary periods of stress tolerance rather than sustained tolerance throughout the stress period. Whether these responses improve plant fitness depends on what happens after this period ends.

## 3 What variation tells us regarding provenance, ecotype, and lineage

Six *Elymus sibiricus* accessions, originating between 3,147 and 4,657 m in altitude, were grown together in a common garden and assessed over 2 years Niu and Qin. Forage and seed yield were associated with different plant traits in structural equation modeling: forage yield increased with leaf width and stem diameter but decreased with plant height, whereas seed yield was associated with leaf length and effective tillering. Accessions from 3,000 m to 4,000 m outperformed those from above 4,000 m, with the highest score from the accession from 3,147 m, close to the elevation of the trial site at 3,156 m. With one accession per source elevation and a single test environment, these results identify promising provenances rather than an optimum, as the authors noted in calling for multi-environment trials. The main result is that forage and seed production are associated with different sets of traits, suggesting that selection targets should differ depending on the production goal.

Double-digest RAD sequencing of 135 *Leymus racemosus* individuals yielded 56,655 SNPs and identified two major genetic lineages associated with the Irtysh and Ulungur basins Zhang et al. Genetic diversity was low, and genetic distance was most strongly correlated with geographic distance. Environmental distance was also strongly correlated with geography but only weakly with genetic distance. Thus, the results are consistent with isolation by distance; however, the independent contribution of the environment remains unresolved (Wang and Bradburd, 2014). The strong heterozygote deficit also warrants caution in attributing low diversity to clonality alone. Nevertheless, the two-lineage structure has clear conservation implications, supporting sampling and *ex situ* conservation across both lineages and their geographic provenance.

## 4 What this Research Topic leaves open

By design, each study in this Research Topic examined the effect of a single stressor, although the natural environments in which these grasses occur expose them to multiple ones. Reservoir drawdown zones, for example, impose annual cycles of drying followed by flooding, while alpine provenances experience together cold temperatures, high light irradiance, and a short season. Responses to combined stresses cannot be predicted from responses to individual stresses (Zandalinas and Mittler, 2022). In C_4_ pasture grasses, for instance, warming can offset the water relations benefit of elevated CO_2_ while increasing the CO_2_-induced decline in forage quality (Habermann et al., 2022). Community-level responses and model-based predictions of productivity were not covered by the four articles in this Research Topic. Experiments that manipulate water and nutrient supply in grass and legume mixtures, or that combine warming with water deficits in forage grasses, indicate how these questions could be tested (Xu et al., 2018; Habermann et al., 2019). Future experiments should test recovery after submergence under different dissolved oxygen conditions, measure whether stay-green plants maintain photosynthetic carbon assimilation under field conditions and conduct multi-site provenance trials under combinations of stresses.

## 5 Concluding remarks

These four studies characterize intraspecific variation at four levels: a leaf-surface trait, a chlorophyll catabolism pathway, a set of provenances, and a pair of genetic lineages. A common limitation is that responses measured in controlled experimental conditions may not predict performance in the field. Rather than claiming that these studies integrate across scales, we consider it more useful to identify this gap and the experiments needed to close it.

## References

[B1] Bailey-Serres J. Lee S. C. Brinton E. (2012). Waterproofing crops: Effective flooding survival strategies. Plant Physiol. 160, 1698–1709. doi: 10.1104/pp.112.208173 23093359 PMC3510103

[B2] Bardgett R. D. Bullock J. M. Lavorel S. Manning P. Schaffner U. Ostle N. . (2021). Combatting global grassland degradation. Nat. Rev. Earth Environ. 2, 720–735. doi: 10.1038/s43017-021-00207-2 37880705

[B3] Broadhurst L. M. Lowe A. Coates D. J. Cunningham S. A. McDonald M. Vesk P. A. . (2008). Seed supply for broadscale restoration: Maximizing evolutionary potential. Evol. Appl. 1, 587–597. doi: 10.1111/j.1752-4571.2008.00045.x 25567799 PMC3352390

[B4] Colmer T. D. Pedersen O. (2008). Underwater photosynthesis and respiration in leaves of submerged wetland plants: Gas films improve CO_2_ and O_2_ exchange. New Phytol. 177, 918–926. doi: 10.1111/j.1469-8137.2007.02318.x 18086222

[B5] Edwards E. J. Osborne C. P. Strömberg C. A. E. Smith S. A. Bond W. J. Christin P.-A. . (2010). The origins of C_4_ grasslands: Integrating evolutionary and ecosystem science. Science 328, 587–591. doi: 10.1126/science.1177216 20431008

[B6] Habermann E. Dias de Oliveira E. A. Contin D. R. Costa J. V. C. P. Costa K. A. P. Martinez C. A. (2022). Warming offsets the benefits of elevated CO_2_ in water relations while amplifies elevated CO_2_-induced reduction in forage nutritional value in the C_4_ grass Megathyrsus maximus. Front. Plant Sci. 13. doi: 10.3389/fpls.2022.1033953 36544868 PMC9760913

[B7] Habermann E. Dias de Oliveira E. A. Contin D. R. Delvecchio G. Viciedo D. O. de Moraes M. A. . (2019). Warming and water deficit impact leaf photosynthesis and decrease forage quality and digestibility of a C_4_ tropical grass. Physiol. Plant 165, 383–402. doi: 10.1111/ppl.12891 30525220

[B8] MacDougall A. S. Vanzant B. Sulik J. Bagchi S. Naidu D. Muraina T. O. . (2026). The global extent of the grassland biome and implications for the terrestrial carbon sink. Nat. Ecol. Evol. 10, 246–257. doi: 10.1038/s41559-025-02955-6 41593186 PMC12929054

[B9] Strömberg C. A. E. (2011). Evolution of grasses and grassland ecosystems. Annu. Rev. Earth Planet. Sci. 39, 517–544. doi: 10.1146/annurev-earth-040809-152402 41139587

[B10] Thomas H. Howarth C. J. (2000). Five ways to stay green. J. Exp. Bot. 51, 329–337. doi: 10.1093/jexbot/51.suppl_1.329 10938840

[B11] Thomas H. Ougham H. (2014). The stay-green trait. J. Exp. Bot. 65, 3889–3900. doi: 10.1093/jxb/eru037 24600017

[B12] Thomas H. Stoddart J. L. (1975). Separation of chlorophyll degradation from other senescence processes in leaves of a mutant genotype of meadow fescue (Festuca pratensis L.). Plant Physiol. 56, 438–441. doi: 10.1104/pp.56.3.438 16659320 PMC541840

[B13] Wang I. J. Bradburd G. S. (2014). Isolation by environment. Mol. Ecol. 23, 5649–5662. doi: 10.1111/mec.12938 25256562

[B14] Winkel A. Visser E. J. W. Colmer T. D. Brodersen K. P. Voesenek L. A. C. J. Sand-Jensen K. . (2016). Leaf gas films, underwater photosynthesis and plant species distributions in a flood gradient. Plant Cell Environ. 39, 1537–1548. doi: 10.1111/pce.12717 26846194

[B15] Xu B. Xu W. Wang Z. Chen Z. Palta J. A. Chen Y. (2018). Accumulation of N and P in the legume Lespedeza davurica in controlled mixtures with the grass Bothriochloa ischaemum under varying water and fertilization conditions. Front. Plant Sci. 9, 165. doi: 10.3389/fpls.2018.00165 29487611 PMC5816928

[B16] Zandalinas S. I. Mittler R. (2022). Plant responses to multifactorial stress combination. New Phytol. 234, 1161–1167. doi: 10.1111/nph.18087 35278228

